# Using Systemic Inflammatory Markers to Predict Microvascular Invasion Before Surgery in Patients With Hepatocellular Carcinoma

**DOI:** 10.3389/fsurg.2022.833779

**Published:** 2022-03-04

**Authors:** Shumin Li, Qianwen Zeng, Ruiming Liang, Jianyan Long, Yihao Liu, Han Xiao, Kaiyu Sun

**Affiliations:** ^1^Department of Gastroenterology and Hepatology, The First Affiliated Hospital, Sun Yat-sen University, Guangzhou, China; ^2^Department of Liver Surgery, The First Affiliated Hospital, Sun Yat-sen University, Guangzhou, China; ^3^Clinical Trials Unit, The First Affiliated Hospital, Sun Yat-sen University, Guangzhou, China; ^4^Division of Interventional Ultrasound, The First Affiliated Hospital, Sun Yat-sen University, Guangzhou, China; ^5^Department of Gastrointestinal Surgery, The First Affiliated Hospital, Sun Yat-sen University, Guangzhou, China

**Keywords:** hepatocellular carcinoma (HCC), microvascular invasion (MVI), inflammatory markers, surgery, predictive model

## Abstract

**Background:**

Mounting studies reveal the relationship between inflammatory markers and post-therapy prognosis. Yet, the role of the systemic inflammatory indices in preoperative microvascular invasion (MVI) prediction for hepatocellular carcinoma (HCC) remains unclear.

**Patients and Methods:**

In this study, data of 1,058 cases of patients with HCC treated in the First Affiliated Hospital of Sun Yat-sen University from February 2002 to May 2018 were collected. Inflammatory factors related to MVI diagnosis in patients with HCC were selected by least absolute shrinkage and selection operator (LASSO) regression analysis and were integrated into an “Inflammatory Score.” A prognostic nomogram model was established by combining the inflammatory score and other independent factors determined by multivariate logistic regression analysis. The receiver operating characteristic (ROC) curve and the area under the curve (AUC) were used to evaluate the predictive efficacy of the model.

**Results:**

Sixteen inflammatory factors, including neutrophil-to-lymphocyte ratio, platelet-to-lymphocyte ratio, etc., were selected by LASSO regression analysis to establish an inflammatory score. Multivariate logistic regression analysis showed that inflammatory score (OR = 2.14, 95% CI: 1.63–2.88, *p* < 0.001), alpha fetoprotein (OR = 2.02, 95% CI: 1.46–2.82, *p* < 0.001), and tumor size (OR = 2.37, 95% CI: 1.70–3.30, *p* < 0.001) were independent factors for MVI. These three factors were then used to establish a nomogram for MVI prediction. The AUC for the training and validation group was 0.72 (95% CI: 0.68–0.76) and 0.72 (95% CI: 0.66–0.78), respectively.

**Conclusion:**

These findings indicated that the model drawn in this study has a high predictive value which is capable to assist the diagnosis of MVI in patients with HCC.

## Introduction

Hepatocellular carcinoma (HCC) is the sixth most common cancer and the third leading cause of cancer-related deaths worldwide, with a recurrence rate up to 70% at 5 years after resection and 15–40% after liver transplantation (1–5).

Microvascular invasion (MVI) is defined as a cluster of tumor cells in the vessels of the surrounding hepatic tissue lined by endothelium, which was visible only under the microscopy (6). Accordingly, the incidence of MVI in HCC is highly variable between studies (ranged from 15 to 57.1%) (7). It has been widely accepted that the presence of MVI is a symbol of aggressive biological behavior and is a critical factor related to high recurrence rate and long term overall survival in patients who undergo resection or liver transplant (7–10). Preoperative diagnosis of MVI can not only offer some guidance for surgeons to determine the surgical plan, but also help physicians select candidates for liver transplantation or adjuvant treatment (7, 11, 12). However, currently, information about MVI mainly comes from postoperative liver specimens. Thus, it is important to be able to predict MVI for patients with HCC before surgery.

A variety of methods have been proposed for the preoperative detection of MVI (13–20). Some studies found that radiological features including tumor size, number of nodules, condition of capsule, intratumoral arteries, and typical dynamic pattern of computed tomography (CT) or contrast-enhanced magnetic resonance (MR) were independently related to the presence of MVI (17–19) and thus, could be useful in MVI diagnosis. Zhang et al. (20) established a nomogram based on the data of 267 patients with HCC and showed that the model incorporating alpha fetoprotein (AFP) level, fusion radiomics features, and arterial peritumoral enhancement achieved a satisfactory prediction of MVI before operation. However, the robustness of models based on radiological features or radiomics features is poor since judging the features and drawing region of interest for feature extraction are highly operator dependent. In addition, the low accuracy of AFP, though widely recognized in the serum test, also limits its application in routine clinical practice. Therefore, a simple and accurate method for preoperative prediction of MVI is warranted.

Inflammatory markers, which are easily obtained by hematological indicators, can reflect systemic inflammatory responses and serve as the surveillance indices for disease progression or therapeutic response (16, 21). It is reported that inflammatory markers can affect tumor development and metastasis by promoting tumor cell proliferation, invasion, and angiogenesis (22). Previous studies have results that showed how some inflammatory markers are the independent factors for MVI (17, 23–26), probably due to potential relationship between systemic inflammatory response and MVI (27, 28). Zeng et al. (29) conducted a meta-analysis and confirmed that there was a significant difference with respect to the neutrophil-to-lymphocyte ratio (NLR) level between an MVI-positive and an MVI-negative group.

However, there are two major pitfalls that commonly exist in these studies. One is the sample size of the studies, which were relatively small (range from 330 to 513) (23, 24, 26). Therefore, conclusion may not be generalized to the whole population. The other is that the models developed in studies only incorporated one or two serum inflammatory markers (17, 23–26). However, different inflammatory markers reflect different aspects of systemic inflammatory response. Therefore, using limited markers may not represent a comprehensive value of inflammatory indices in predicting MVI. Therefore, in this study, we constructed an MVI prediction model based on a large sample data and integrated inflammatory indices and tried to clarify the correlation between inflammatory markers and MVI.

## Patients and Methods

### Patient Selection

We retrospectively collected the data of 2,690 patients with HCC who initially underwent liver resection in the First Affiliated Hospital of Sun Yat-sen University between February 2002 and May 2018. The inclusion criteria were as follows: (a) primary HCC diagnosed by pathological examination without previous treatment; and (b) has available pathological data of presence or absence of MVI. The exclusion criteria were as follows: (a) diagnosed with other cancers; (b) with macrovascular invasion confirmed by radiological or pathological examination; and (c) without complete clinical data. Ultimately, 1,058 patients were enrolled in this study. Informed consent was waived and the study was approved by the Institutional Review Board of The First Affiliated Hospital of Sun Yat-sen University.

### Data Collection

Demographic variables, including age and gender, were collected. Latest results of laboratory examination (usually 1–3 days before surgery) included AFP, hepatitis B surface antigen (HBsAg), white blood cell (WBC), neutrophil (NE), monocyte (MO), lymphocyte (LY), platelet (PLT), albumin (ALB), aspartate transaminase (AST), alanine transaminase (ALT), gamma-glutamyl transpeptidase (GGT), hemoglobin (HB), NLR, lymphocyte-to-monocyte ratio (LMR), platelet-to-lymphocyte ratio (PLR), neutrophil-to-mononcyte-plus-lymphocyte ratio (NMLR), aspartate transaminase-to-neutrophil ratio index (ANRI), prognostic nutritional index (PNI), aspartate transaminase-to-platelet ratio index (APRI), gamma-glutamyl transpeptidase-to-platelet ratio (GPR), fibrosis index based on 4 factors (FIB-4), and albumin-to-gamma-glutamyl transpeptidase (AGR). NLR, LMR, PLR, NMLR, ANRI, APRI, PNI, GPR, FIB-4, and AGR were calculated by formulae listed in the reference table (details were shown in Supplementary Material). The presence or absence of hepatitis C, varicose veins, splenauxe, ascites, and liver cirrhosis were also obtained. Portal hypertension (PHT) was defined as esophageal varices or splenauxe related to the PLT counts lower than 100 × 10^9^/L (30). The Barcelona Clinic Liver Cancer (BCLC) stage, TNM stage, and Child-pugh grade were determined based on the radiology and laboratory findings. Tumor size and tumor number were evaluated on CT or MR by two radiologists with more than 5 years of experience in HCC radiology.

### Statistical Analysis

Patients meeting the inclusion criteria were randomly divided into the training group (*n* = 740) and the validation group (*n* = 318) by the ratio of 7:3. Categorical variables were described as number and percentage, and continuous variables were presented as mean ± standard deviation or median and interquartile range. Comparison between the training group and validation group were performed by the *t*-test or Mann–Whitney *U*-test for continuous variables and chi-square test or Fisher exact test for categorical variables.

The relationship between preoperative characteristics and MVI was analyzed using univariate logistic regression analysis for categorical variables and least absolute shrinkage and selection operator (LASSO) regression analysis for continuous variables. In order to comprehensively and systematically explain the role of inflammatory markers, all the inflammatory markers selected by the LASSO regression were included in the multivariate regression analyses to calculate coefficients and integrated into an “inflammatory score” according to the following formula: inflammatory score = $\sum_{i=1}^{n}Xi$, where *X* is the selected marker and n is the number of markers (details were shown in Supplementary Formula). Then, categorical variables with statistical significance together with age and inflammatory score were included in stepwise logistic regression analysis. Subsequently, the MVI prediction model was built based on the independent MVI predictors sifted by the stepwise multivariate logistic regression at a significant level. A nomogram was ultimately constructed on the MVI prediction model as a graphical presentation. The receiver operating characteristic (ROC) curve was plotted and the area under the curve (AUC) was calculated for the evaluation of model in both the training and the validation group. The “Optimal Cut points” package was used for ROC analysis to determine optimal cut-off value. Calibration curves were generated in both the training group and validation group to measure the diagnostic performance of the model. The accuracy, sensitivity, specificity, positive predictive value, and negative predictive value used to differentiate between the absence and presence of MVI were also calculated. Statistical significance was considered as a two-tailed *p*-value of < 0.05. All statistical analyses were performed by SAS version 9.4 software (SAS Institute, Cary, NC) and R 3.6.0.

## Results

### Patient Characteristics

A total of 1,058 patients with HCC, 430 of whom (40.6%) were diagnosed with MVI by histological examination, were enrolled in this retrospective study. Of this cohort, 928 (87.7%) were males and 130 (12.3%) were females. The median age of all patients was 54.1 years (range, 44.9–61.7 years). Patients were randomly divided into the training group (*n* = 740) and the validation group (*n* = 318) by the ratio of 7:3, and the baseline characteristics are shown in Table 1. There was no significant difference between the training group and validation group in regard to the distribution of characteristics (*p* = 0.10–0.89), with the exception of PLT (*p* < 0.001), PLR (*p* = 0.006), APRI (*p* = 0.01), and FIB-4 (*p* = 0.02).

**Table 1:** Comparison of patients' characteristics in the training and validation cohorts.

**Variables**		**Training cohort (*n* = 740)**	**Validation cohort (*n* = 318)**	***P*-value**
Gender	Male	651 (88)	277 (87.1)	0.69
	Female	89 (12)	41 (12.9)	
Age (years)		54.4 (44.7–61.7)	53.7 (45.2–61.7)	0.89
Hepatitis C	Present	16 (2.2)	9 (2.8)	0.51
	Absent	724 (97.8)	309 (97.2)	
HBsAg	Positive	626 (84.6)	281 (88.4)	0.11
	Negative	114 (15.4)	37 (11.6)	
AFP (ng/mL)	<20	292 (39.5)	130 (40.9)	0.67
	≥20	448 (60.5)	188 (59.1)	
WBC (10^9^/L)		5.8 (4.9–7.3)	5.8 (4.9–7.3)	0.38
NE (10^9^/L)*		0.6 (0.1)	0.6 (0.1)	0.31
MO (10^9^/L)		0.1 (0.1–0.1)	0.1 (0.1–0.1)	0.79
LY (10^9^/L)		0.3 (0.3–0.4)	0.3 (0.3–0.4)	0.28
PLT (10^9^/L)		183.5 (144.0–232.5)	164.0 (126.0–208.0)	<0.001
ALB (g/L)		39.8 (36.9–42.3)	39.6 (37.0–42.4)	0.84
ALT (U/L)		33.0 (23.0–48.0)	33.0 (24.0–50.0)	0.84
AST (U/L)		34.0 (26.1–50.0)	35.0 (27.0–49.0)	0.47
GGT (U/L)		56.0 (36.0–100.0)	54.0 (33.0–100.0)	0.52
NLR		1.9 (1.4–2.6)	1.8 (1.3–2.5)	0.32
LMR		3.7 (2.7–4.9)	3.8 (2.9–4.8)	0.35
PLR		99.0 (75.1–136.0)	95.4 (67.9–125.4)	0.006
NMLR		2.2 (1.7–2.9)	2.1 (1.6–2.8)	0.24
ANRI		26.8 (18.6–42.4)	28.7 (17.9–44.6)	0.32
APRI		0.5 (0.4–0.8)	0.6 (0.4–0.9)	0.01
PNI		70.3 (53.6–88.8)	71.9 (55.0–90.1)	0.75
FIB-4		1.8 (1.3–2.7)	2.0 (1.3–3.1)	0.02
GPR		0.7 (0.4–1.3)	0.7 (0.4–1.3)	0.33
AGR		0.7 (0.4–1.1)	0.7 (0.4–1.2)	0.53
Tumor size (cm)	<5	373 (50.4)	166 (52.2)	0.59
	≥5	367 (49.6)	152 (47.8)	
Tumor number	1	582 (78.6)	237 (74.5)	0.14
	>1	158 (21.4)	81 (25.5)	
Child-pugh grade	A	703 (95.0)	303 (95.3)	0.85
	B	37 (5.0)	15 (4.7)	
BCLC stage	0–A	566 (76.5)	235 (73.9)	0.37
	B–D	174 (23.5)	83 (26.1)	
TNM stage	T1	557 (75.3)	224 (70.4)	0.10
	T2–T4	183 (24.7)	94 (29.6)	
Varicose veins	Present	61 (8.2)	28 (8.8)	0.76
	Absent	679 (91.8)	290 (91.2)	
Splenauxe	Present	232 (31.4)	107 (33.6)	0.46
	Absent	508 (68.6)	211 (66.4)	
Liver cirrhosis	Present	366 (49.5)	160 (50.3)	0.80
	Absent	374 (50.5)	158 (49.7)	
Ascites	Present	38 (5.1)	17 (5.3)	0.89
	Absent	702 (94.9)	301 (94.7)	
PHT	Present	94 (12.7)	47 (14.8)	0.36
	Absent	646 (87.3)	271 (85.2)	

Unless otherwise indicated, categorical variables are described as numbers (percentage) and continuous variables are presented as median (interquartile range). *Data are shown as mean (standard deviation).

*HBsAg, hepatitis B surface antigen; AFP, alpha fetoprotein; WBC, white blood cell; NE, neutrophil; MO, monocyte; LY, lymphocyte; PLT, platelet; ALB, albumin; AST, aspartate transaminase; ALT, alanine transaminase; GGT, gamma-glutamyl transpeptidase; NLR, neutrophil-to-lymphocyte ratio; LMR, lymphocyte-to-monocyte ratio; PLR, platelet-to-lymphocyte ratio; NMLR, neutrophil-to-mononcyte-plus-lymphocyte ratio; ANRI, aspartate transaminase-to-neutrophil ratio index; APRI, aspartate transaminase-to-platelet ratio index; PNI, prognostic nutritional index; FIB-4, fibrosis index based on four factors; GPR, gamma-glutamyl transpeptidase-to-platelet ratio; AGR, albumin-to-gamma-glutamyl transpeptidase; BCLC, Barcelona Clinic Liver Cancer; PHT, portal hypertension*.

### Inflammatory Score

**Figure 1:**
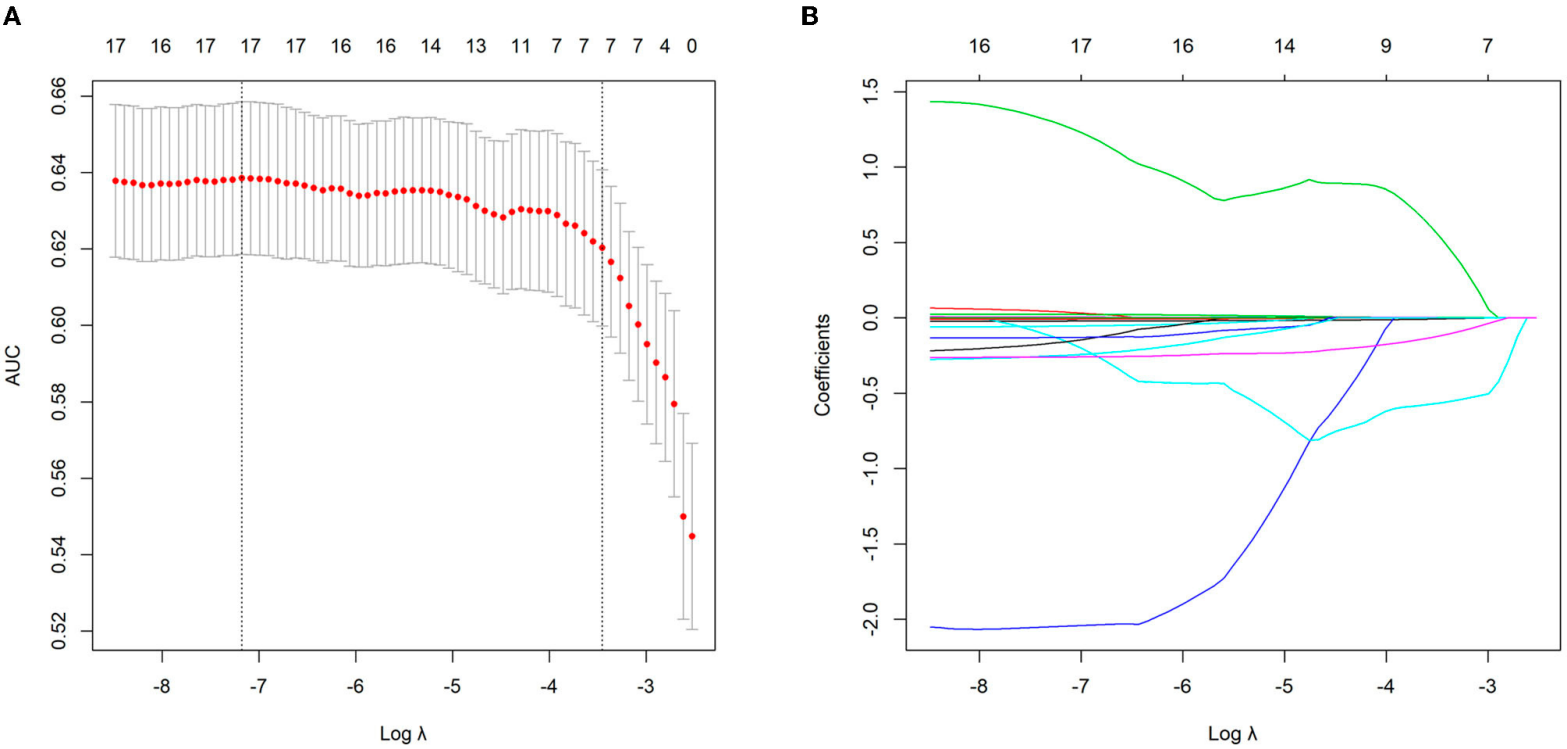
Continuous variables selection using the least absolute shrinkage and selection operator (LASSO) logistic regression model in the training group. **(A)** The area under the curve (AUC) was plotted vs. log (Lambda). Dotted vertical lines indicate the lambda value with the minimum error and the 1 standard error of the minimum criteria. **(B)** LASSO coefficient profiles of the continuous variables.

Among 20 continuous variables, 17 (age, WBC, NE, MO, LY, PLT, ALB, ALT, AST, GGT, LMR, PLR, NMLR, ANRI, APRI, GPR, and AGR) were selected by the LASSO regression based on the data of the training group (Figure 1). Of these 17 variables, 16 were inflammatory markers and were then integrated into an inflammatory score (details were shown in “Patients and Methods” section and Supplementary Formula). The inflammatory score for each patient in the training and validation groups was calculated using the formula described above.

### Univariate and Multivariate Analysis of MVI-Related Predictors

Univariate logistic regression analysis for categorical variables showed that AFP (*p* < 0.001), tumor size (*p* < 0.001), and TNM stage (*p* = 0.03) were potential risk factors for the presence of MVI (data not shown). Subsequently, stepwise multivariate analysis was employed to screen the potential risk factors sifted by univariate logistic regression and LASSO regression (age and inflammatory score). Results revealed that inflammatory score (OR = 2.14, 95% CI: 1.63–2.88, *p* < 0.001), AFP (OR = 2.02, 95% CI: 1.46–2.82, *p* < 0.001), and tumor size (OR = 2.37, 95% CI: 1.70–3.30, *p* < 0.001) were independent factors for MVI (Table 2).

**Table 2:** Multivariate logistic regression analysis of independent risk factors for microvascular invation (MVI) in the training cohort.

**Variables**	**OR (95% CI)**	***P*-value**
Inflammatory score	2.14 (1.63–2.88)	<0.001
AFP (ng/mL), <20 vs. ≥20	2.02 (1.46–2.82)	<0.001
Tumor size (cm), <5 vs. ≥5	2.37 (1.70–3.30)	<0.001

*MVI, microvascular invasion; OR, odds ratio; CI, confidence interval; AFP, alpha fetoprotein*.

### Development and Validation of a Nomogram for MVI Prediction

**Figure 2:**
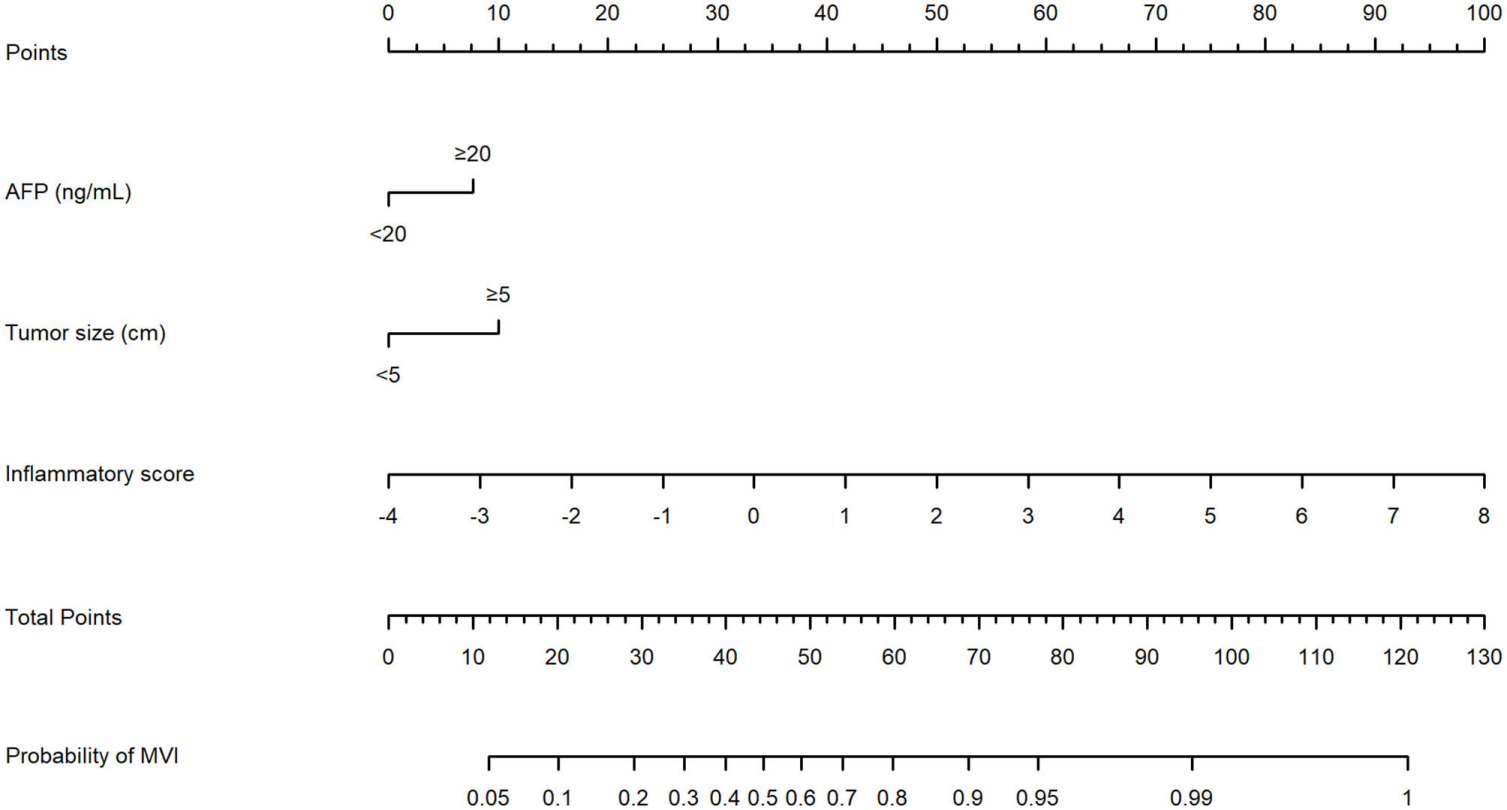
The nomogram model established by incorporating the alpha fetoprotein (AFP), tumor size, and inflammatory score to predict the risk of microvascular invasion (MVI) for patients with hepatocellular carcinoma (HCC).

**Figure 3:**
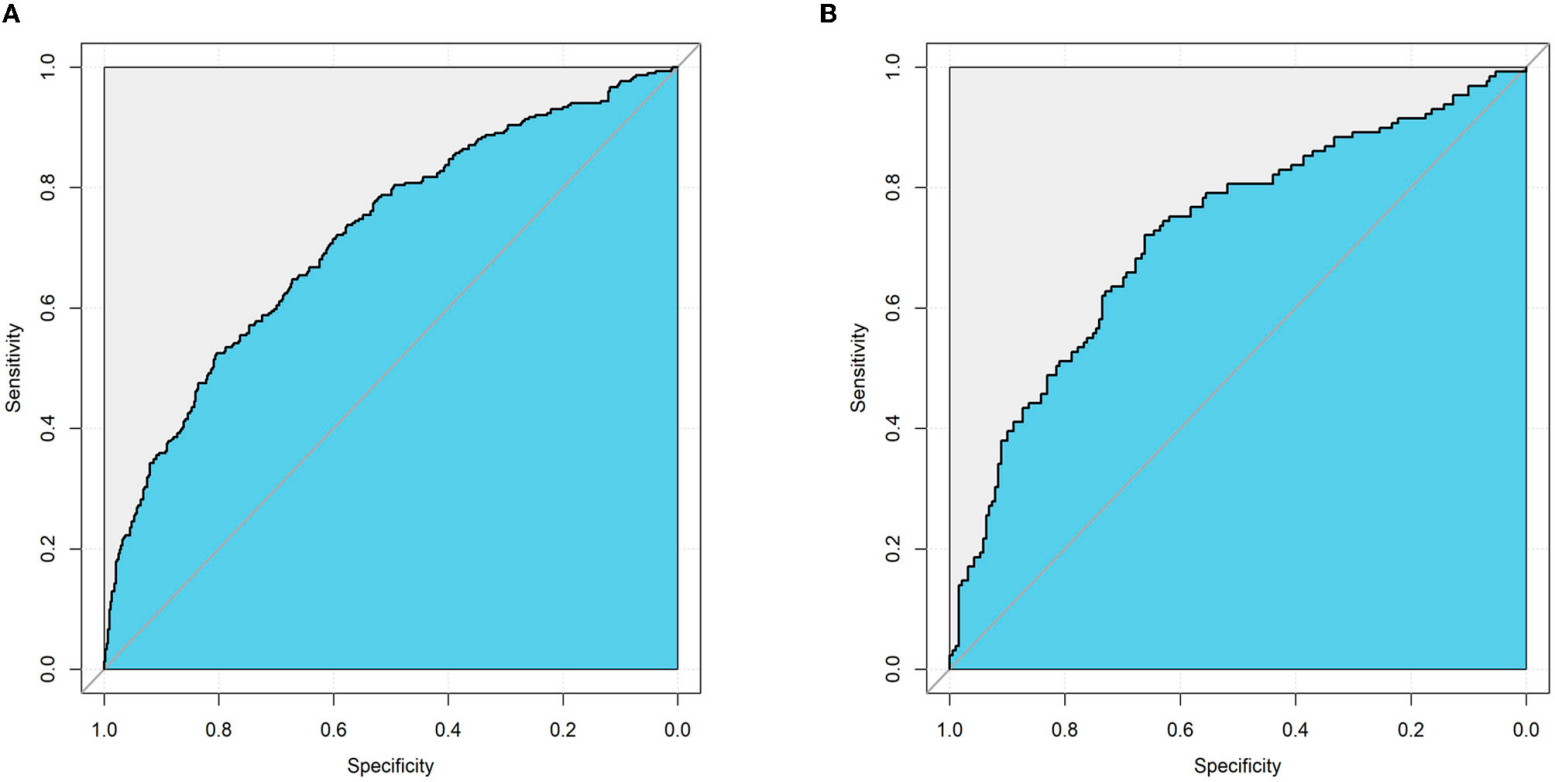
The performance of the nomogram model for predicting MVI by receiver operating characteristic (ROC) curve. **(A)** ROC curve in the training cohort. **(B)** ROC curve in the validation cohort.

**Figure 4:**
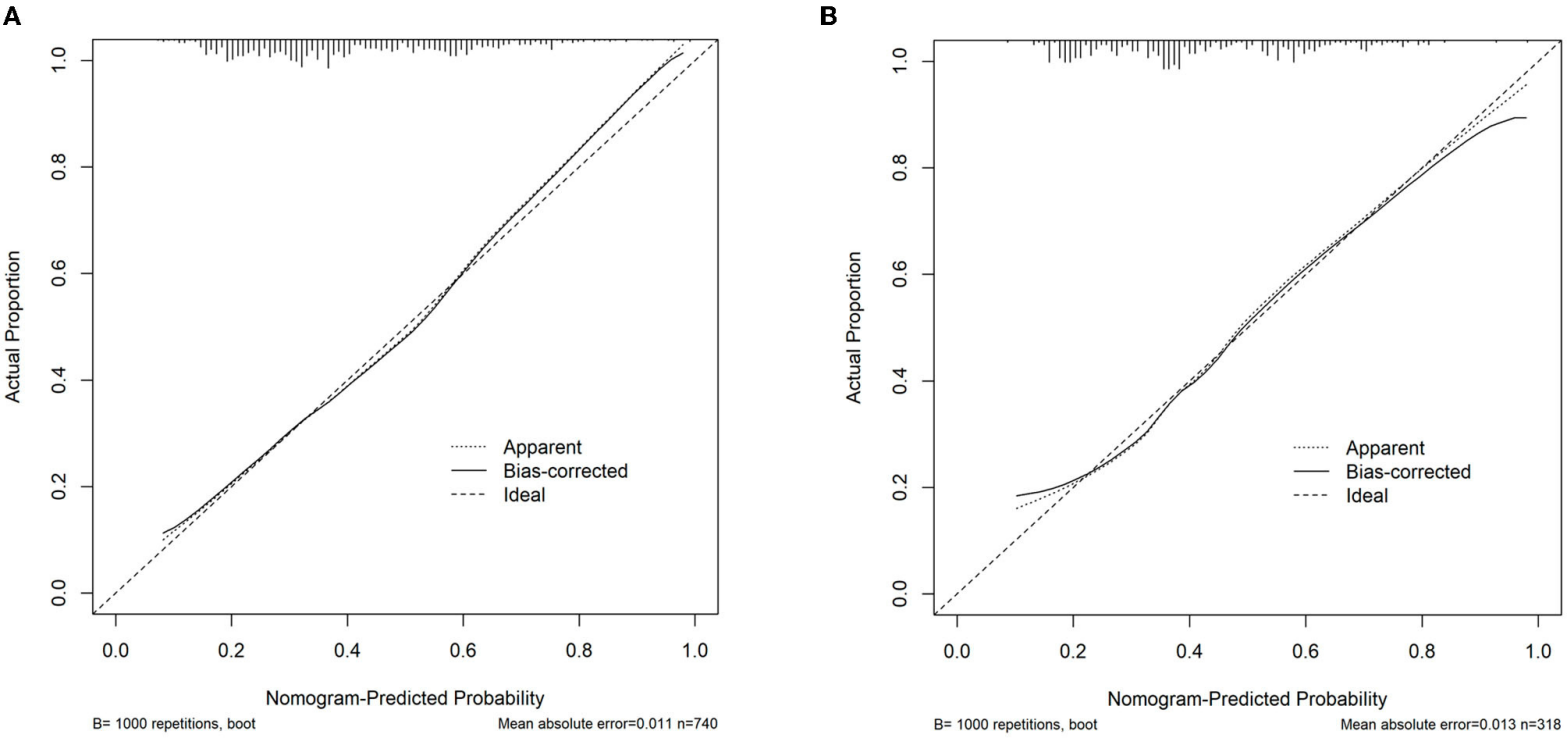
Assessment of the nomogram model calibration. **(A)** Calibration curve in the training cohort. **(B)** Calibration curve in the validation cohort.

An MVI prediction model incorporating inflammatory score, AFP, and tumor size sifted by logistic regression was eventually constructed. To put the prediction model into clinical use, we built a nomogram based on this model as a graphical presentation (Figure 2). The nomogram exhibited an AUC of 0.72 (95% CI: 0.68–0.76) in the training group and 0.72 (95% CI: 0.66–0.78) in the validation group (Figure 3). Calibration curves indicated a good agreement between the predicted probability of the model and actual MVI evaluation in both groups (Figure 4). In addition, the model showed that based on the cut-off value of predictive probability set at 0.38, the accuracy, sensitivity, specificity, positive predictive value, and negative predictive value used to distinguish the presence or absence of MVI were 65.5, 65.4, 65.6, 56.6, and 73.5%, respectively, in the training group and 67.3, 63.6, 69.8, 59.0, and 73.7%, respectively, in the validation group (Table 3).

**Table 3:** Accuracy of the nomogram in predicting MVI.

**Index**	**Training group (*n* = 740)**	**Validation group (*n* = 318)**
AUC (95% CI)	0.72 (0.68–0.76)	0.72 (0.66–0.78)
Sensitivity	65.4%	63.6%
Specificity	65.6%	69.8%
Positive predictive value	56.6%	59.0%
Negative predictive value	73.5%	73.7%
Accuracy	65.5%	67.3%

*MVI, microvascular invasion; AUC, area under the curve; CI, confidence interval*.

## Discussion

In this study, we established an inflammatory score combining 16 inflammatory markers and demonstrated them to be an independent factor for MVI. A nomogram was then developed based on inflammatory score, AFP, and tumor size for patients with HCC to identify MVI before surgery. The nomogram achieved satisfying performance and was convenient to use for its easily available preoperative clinical data.

Microvascular invasion (MVI) remains a risk factor for tumor recurrence and metastasis in hepatocellular carcinoma (HCC) (7–10), with a great impact on guiding clinical decisions (7, 11, 12). Yet, a simple and precise method for preoperative MVI prediction is unreached. Previous studies have made progress in constructing models based on radiological features or radiomic features for MVI prediction (18–20, 31) Xu et al. (18) used the data of 495 patients with HCC who underwent preoperative contrast-enhanced CT to develop a risk model based on MVI-related radiomic scores and clinico-radiologic factors for MVI prediction. The model revealed an MVI identification rate of 88% with a specificity of 76.8–79.2%. Li et al. found that the nomogram combined with clinico-radiologic factors and the fusion radiomics signatures of hepatobiliary phase reached a high accuracy of 87.7% in the training cohort and 83.9% in the validation cohort for predicting MVI preoperatively (31). However, the identification of most imaging features lacks a unified standard and depends on the experience and subjective diagnosis of radiologists. In addition, some imaging parameters are even too sophisticated to be grasped by clinicians. Therefore, whether these models can be put into routine clinical use is still a question to be answered. In contrast, serum indices, which are easily obtained, can achieve the unification and standardization of indicators and make the data of various institutions comparable. Thus, it can serve as a supplementary method for diseases diagnosis in the areas lacking advanced imaging equipment or well-experienced radiologists.

As the indicators, such as liver function and blood routine test, are easy to obtain by routine preoperative hematological examination, inflammatory markers have been proven to be the prognostic factors in HCC (27, 32). As for their roles in preoperative prediction, previous studies have incorporated these indices into the nomogram for predicting MVI and have made some progress (17, 23–26) Deng et al. (24) showed that NLR was a useful biomarker for MVI prediction based on the data of 513 patients with HCC. Another study found that the derived NLR and LMR were identified as the significant predictors for MVI (25). Nevertheless, those studies included only one or two inflammatory markers in the model and thus may not represent comprehensive value of inflammatory indices in predicting MVI. In this study, to retain the information of inflammatory markers as much as possible and reduce the complexity of the model, a total of 16 inflammatory indices were screened out and were then synthesized into an inflammatory score which proved to be an independent risk factor for MVI. Although there is no direct evidence to clarify the association between inflammatory markers and the presence of MVI, Motomura et al. (33) found that upregulated level of IL-7, a pro-angiogenic cytokine, was associated with a higher NLR score. Another study showed that some cytokines that are produced in cancer, when acting on platelets, can lead to the release of platelet-secreted mediators such as vascular endothelial growth factor and platelet derived growth factor, which can play role in angiogenesis (34). In summary, our results underline the comprehensive and great value of inflammatory markers used in predicting MVI.

In order to better identify MVI preoperatively, other easily available clinical indicators, termed tumor size and AFP, were also incorporated. Various studies have demonstrated tumor size to be independent predictors for MVI (13, 17, 23), which is also confirmed in this study. Pawlik et al. (35) found that the incidence of MVI in patients with HCC significantly elevated with the increase of tumor size (risk of MVI could up to 55% when the tumor was larger than 5 cm). The current study set a cut-off value of tumor size in 5 cm and revealed that it could be applied to MVI risk stratification. On the other hand, AFP has also been widely proved to be independent risk factor for MVI (13, 17, 23, 24). Our results confirmed the same tendency of AFP concentrations to elevate with the risk of MVI (36). In a word, this study showed a good performance of tumor size, AFP, and inflammatory markers in predicting MVI.

In this study, a nomogram, based on the large sample size data, was built on the preoperative available data of inflammatory score, AFP, and tumor size. According to the cut-off value, patients can be separated into a high-risk or low-risk subgroup of MVI presence, which may play a role in guiding the clinical decision. In addition, because the indicators included in the model are easy to obtain and be understood by clinicians, the model is able to serve as a good auxiliary method to preoperatively identify MVI for patients with HCC in various regions with no limitation of clinicians' experience.

This study exists with several limitations. First, our study is a single-center and retrospective study without external validation. Thus, a multi-center and prospective study is needed to verify the reliability of the model. Second, it should be noted that the level of inflammatory markers can abnormally rise in several conditions, such as infection, injury, or benign hepatic disease. Clinically, these serum indicators can also be affected by the time they are checked, patients' diet, and their mental state. Therefore, how to avoid or control the influence of these confounding factors on inflammatory indicators remains a challenge.

## Conclusion

In conclusion, we established an inflammatory score that integrates several inflammatory markers and found an independent risk factor for MVI. We then combined the inflammatory score with AFP and tumor size to build an MVI prediction model. The model revealed high predictive value and may be applied to routine clinical use to guide the decision-making of treatments for patients with HCC.

## Data Availability Statement

The raw data supporting the conclusions of this article will be made available by the authors, without undue reservation.

## Ethics Statement

The studies involving human participants were reviewed and approved by the Institutional Review Board of the First Affiliated Hospital of Sun Yat-sen University. The patients/participants provided their written informed consent to participate in this study.

## Author Contributions

SL, QZ, RL, JL, and YL conceived the study and collected data. RL, JL, and YL analyzed the data. SL and QZ drafted the manuscript. HX and KS helped to revise the manuscript. All authors contributed to the article and approved the submitted version.

## Funding

This present work was supported by grants from the Guangdong Basic and Applied Basic Research Foundation (No. 2019A1515111168) and the Natural Science Foundation of Guangdong Province, China (No. 2018A030310326).

## Conflict of Interest

The authors declare that the research was conducted in the absence of any commercial or financial relationships that could be construed as a potential conflict of interest.

## Publisher's Note

All claims expressed in this article are solely those of the authors and do not necessarily represent those of their affiliated organizations, or those of the publisher, the editors and the reviewers. Any product that may be evaluated in this article, or claim that may be made by its manufacturer, is not guaranteed or endorsed by the publisher.
