# Supplementary material for: Using Systemic Inflammatory Markers to Predict Microvascular Invasion Before Surgery in Patients With Hepatocellular Carcinoma
Source: Front Surg. 2022 Mar 4;9:833779. doi: 10.3389/fsurg.2022.833779 (PMC8931769; doi:10.3389/fsurg.2022.833779)
Supplement: Supplementary file 1 [file Data_Sheet_1.docx]

Supplementary Material

# Reference Table

| Variables | Calculating formulae |
| --- | --- |
| NLR | NLR = NE counts (10^9^/L)/LY counts (10^9^/L) |
| LMR | LMR = LY counts (10^9^/L)/MO counts (10^9^/L) |
| PLR | PLR = PLT counts (10^9^/L)/LY counts (10^9^/L) |
| NMLR | NMLR = (NE counts (10^9^/L) + MO counts (10^9^/L))/LY counts (10^9^/L) |
| ANRI | ANRI = [(AST level (U/L)/its upper limit of normal (ULN))/NE counts (10^9^/L)] × 100 |
| APRI | APRI = [(AST level (U/L)/its ULN)/PLT counts (10^9^/L)] × 100 |
| PNI | PNI = ALB level (g/L) × LY counts (10^9^/L) |
| GPR | GPR = [(GGT level (U/L)/its ULN)/PLT counts (10^9^/L)] × 100 |
| FIB-4 | FIB-4 = age (years) × AST level (U/L)/[(PLT counts (10^9^/L) × (ALT level (U/L)) ^1/2^] |
| AGR | AGR = ALB level (g/L)/GGT level (U/L) |

**Abbreviations:** NLR, neutrophil-to-lymphocyte ratio; LMR, lymphocyte-to-monocyte ratio; PLR, platelet-to-lymphocyte ratio; NMLR, neutrophil-to-mononcyte-plus-lymphocyte ratio; ANRI, aspartate transaminase-to-neutrophil ratio index; APRI, aspartate transaminase-to-platelet ratio index; PNI, prognostic nutritional index; GPR, gamma-glutamyl transpeptidase-to-platelet ratio; FIB-4, fibrosis index based on 4 factors; AGR, albumin-to-gamma-glutamyl transpeptidase.

# Supplementary Formula

Inflammatory score = -0.88956 + 0.05796 × WBC counts (10^9^/L) + 1.24947 × NE counts (10^9^/L) - 2.25990 × MO counts (10^9^/L) + 0.00677 × LY counts (10^9^/L) - 0.00412 × PLT counts (10^9^/L) + 0.00310 × ALB level (g/L) - 0.00778 × ALT level (U/L) + 0.02523 × AST level (U/L) + 0.00400 × GGT level (U/L) - 0.06092 × LMR + 0.00653 × PLR - 0.17387 × NMLR - 0.00698 × ANRI - 0.19101 × APRI - 0.26722 × GPR - 0.26030 × AGR
